# Life History Traits in Two *Drosophila* Species Differently Affected by Microbiota Diversity under Lead Exposure

**DOI:** 10.3390/insects12121122

**Published:** 2021-12-15

**Authors:** Mirjana Beribaka, Mihailo Jelić, Marija Tanasković, Cvijeta Lazić, Marina Stamenković-Radak

**Affiliations:** 1 Faculty of Technology Zvornik, University of East Sarajevo, Karakaj 34A, 75400 Zvornik, Bosnia and Herzegovina; ceca.lazic96@hotmail.com; 2 Faculty of Biology, University of Belgrade, Studentski trg 16, 11000 Belgrade, Serbia; mihailoj@bio.bg.ac.rs (M.J.); marina@bio.bg.ac.rs (M.S.-R.); 3 Department of Genetics of Populations and Ecogenotoxicology, Institute for Biological Research “Siniša Stanković”—National Institute of the Republic of Serbia, University of Belgrade, Bulevar Despota Stefana 142, 11060 Belgrade, Serbia; marija.tanaskovic@ibiss.bg.ac.rs

**Keywords:** *Drosophila melanogaster*, *Drosophila subobscura*, egg-to-adult viability, developmental time, microbiota diversity, lead exposure

## Abstract

**Simple Summary:**

Microbiota have a significant functional role in the life of the host, including immunity, lifespan and reproduction. *Drosophila* species are attractive model organisms for investigating microbiota diversity from different aspects due to their simple gut microbiota, short generation time and high fertility. Considering such an important role of the microbiota in the life of *Drosophila*, we investigated the extent to which lead (Pb), as one of the most abundant heavy metals in the environment, affects the microbiota and the fitness of this insect host. The results indicate that different factors, such as population origin and sex, may affect individual traits differently and this could be species-specific. In addition, there are members of microbiota that help the host to overcome environmental stress and they could play a key role in reducing the fitness cost in such situations. Studying the influence of microbiota on the adaptive response to heavy metals and the potential implications on overall host fitness is of great pertinence.

**Abstract:**

Life history traits determine the persistence and reproduction of each species. Factors that can affect life history traits are numerous and can be of different origin. We investigated the influence of population origin and heavy metal exposure on microbiota diversity and two life history traits, egg-to-adult viability and developmental time, in *Drosophila melanogaster* and *Drosophila subobscura,* grown in the laboratory on a lead (II) acetate-saturated substrate. We used 24 samples, 8 larval and 16 adult samples (two species × two substrates × two populations × two sexes). The composition of microbiota was determined by sequencing (NGS) of the V3–V4 variable regions of the 16S rRNA gene. The population origin showed a significant influence on life history traits, though each trait in the two species was affected differentially. Reduced viability in *D. melanogaster* could be a cost of fast development, decrease in *Lactobacillus* abundance and the presence of *Wolbachia*. The heavy metal exposure in *D. subobscura* caused shifts in developmental time but maintained the egg-to-adult viability at a similar level. Microbiota diversity indicated that the *Komagataeibacter* could be a valuable member of *D. subobscura* microbiota in overcoming the environmental stress. Research on the impact of microbiota on the adaptive response to heavy metals and consequently the potential tradeoffs among different life history traits is of great importance in evolutionary research.

## 1. Introduction

The intestines of animals are occupied by diverse communities of microorganisms that can affect different aspects of host health. The microbiota plays a key role in many aspects of host life, including development, digestion, behavior and the immune system [1,2,3]. Simple gut microbiota of *Drosophila* species, short generation time and high fecundity are some of the reasons that make them an attractive model for studying the significance of gut microbiome from different aspects. *Drosophila* hosts only a small number of bacterial populations in its gut, but includes species present in the human microbiota as well. *Drosophila* gut microbiota in laboratory is represented by a low-diversity bacterial community [4,5], but it has great implications on its overall health. The gut microbiome of *Drosophila* contributes to a variety of host traits, such as innate immunity [6], lifespan [7,8,9], nutrition and reproduction [10] and behavior [11,12]. Shifts in microbiota could lead to serious consequences on host physiology, causing even mortality [13]. Thus, it is important to investigate the factors that shape the composition and diversity of microbiota and their possible implications on the host.

One of the greatest problems that animals currently face within the natural environment is pollution. Due to the anthropogenic factors, the presence of pollutants is widespread in the air, soils and water. Heavy metals are pollutants commonly found in nature, with great impact on plants and animals. Recently, it has been reported that heavy metals, such as arsenic, cadmium, lead and mercury, have detrimental effects on the diversity of terrestrial invertebrates at levels below those considered safe for humans [14]. The population-level phenotypic variability of different species depends on the interaction between genetic and environmental variability. The variability in the components of adaptive value (fitness) is an aspect of phenotypic variability. Lead is one of the widespread heavy metals that has previously been reported to have a major negative impact on *Drosophila* fitness [15]. However, it has been shown that its negative impact can be modified depending on the population genetic background [16], genome heterozygosity [17] and genetic variation [18]. Since *Drosophila* species are mainly exposed to lead through food intake, it was suggested that gut microbiota could also have an impact on the species resistance to lead toxicity. Our previous research suggested that bacterial diversity increased in two *Drosophila* species after extended exposure to a lead-saturated substrate. This increase in bacterial diversity underlined certain bacterial genera, such as *Komagataeibacter* and *Acetobacter*, that could be good lead-tolerant members of microbiota [5].

The results obtained in our previous study suggested the difference in shifts of microbiota composition between natural populations of two *Drosophila* species under laboratory conditions on standard and lead-saturated substrate after 13 generations [5]. In the present paper we investigate the microbiota of the same lab-reared *Drosophila* species (*D. melanogaster* and *D. subobscura*), but from each of the two distinct localities on standard and lead-saturated substrate after an additional 22 generations (35 in total). Further, we explore if the changes in life history traits (egg-to-adult viability and developmental time) are associated with microbiota content enabling different responses to lead (Pb) in those experimental groups. The influence of lead exposure was discussed for the population, species, substrate and sex levels for both microbiota and fitness components. The microbiota was analyzed using NGS sequencing of the V3-V4 16S rRNA gene and assessed through diversity indices and taxonomical analysis.

The aim of this study was to determine the impact of prolonged lead exposure in two lab-reared *Drosophila* species, each from two populations on microbiota composition and life history traits, to find potential cause-and-effect relationships between them and to differentiate the response of different origin, species and sex to stress factors.

## 2. Materials and Methods

### 2.1. Sample Collection and Laboratory Maintenance

In this study, both species *(D. melanogaster* and *D. subobscura*) were sampled from two localities in Serbia: Kalna (43.4217 N, 22.4159 E) and Slankamen (45.1415 N, 20.2586 E). Flies of both species were collected with a sweeping net using fermented apple traps. They were maintained in the laboratory at 19 ± 0.5 °C, 12 h:12 h light-dark cycle in 240 mL bottles containing 40 mL of the control substrate (standard-St) or the Pb-acetate-saturated substrate (labeled as C3). The control substrate consisted of standard molasses corn meal diet (14 g agar, 208 g corn meal, 188 g sugar, 40 g dry active yeast, 5 g Nipagin diluted in 60 mL of 96% ethanol in 2.2 L distilled water) and the Pb-acetate-saturated substrate contained 1000 μg/mL of lead acetate. The standard substrate flies were grown originally for 45 and 30 generations (*D. subobscura* and *D. melanogaster*, respectively). After that, they were maintained for another 35 generations on the standard substrate and the Pb-acetate-saturated substrate. Both species were maintained at 19 °C; *D. melanogaster* is successfully reared at 19–25 °C, while for *D. subobscura* 19 °C is the optimal temperature. Since *D. subobscura* generally does not lay eggs in laboratory conditions without dry yeast powder, the powder was added to the substrate surface in both species in order to maintain equal conditions (detailed description is given in Beribaka et al. [5]). For the same purposes, the Nipagin, which was reported to modify the microbiota associated with the flies to some extent, was added in all substrates in the same amount at the same time [19].

### 2.2. Experimental Setup

On the first day of the experiment, 30 pairs of flies per bottle were transferred to a fresh substrate for 5 days; they were then removed from the bottles. After eclosion started, virgin males and females of *D. melanogaster* were collected every 12 h and once every 24 h for *D. subobscura* and separated by sex. Bottles with eclosing *D. subobscura* were additionally held in the dark to avoid possible mating. When 150 virgin males and females from each group were collected, 30 females and 30 males per bottle (5 bottles per group) within each group were placed to mate for 3 days. After that, the bottles were covered with Petri dishes that contained substrate and yeast dissolved in distilled water on the substrate surface and turned upside-down to enable flies to lay eggs. The eggs were collected every 8 h from the Petri dishes and transferred into 50 mL vials with 15 mL of the substrate and 1-2 drops of dissolved yeast added to the surface. For each group, 30 vials with 30 eggs were established (additional 5 vials per group were added for larvae collection). The F1 generation eclosion was recorded every 24 h until there were no new individuals in the vial for a period of 72 h; the flies were then stored in EtOH for NGS (separated by sex) at −20 °C. The egg-to-adult viability was calculated per vial as the percentage of individuals that emerged from the 30 eggs. The egg-to-adult developmental time was calculated using the formula:

(1) $$DT=\frac{\Sigma n_{d}*d}{\Sigma n_{d}}$$

where *n_d_* is the number of flies emerging in *d* days after the eggs were laid.

The third instar larvae were collected from the additional 5 vials per group and after 64 h of incubation they were rinsed with distilled water and stored in EtOH at −20 °C for NGS.

The samples were labeled as follows: *D. melanogaster*—Dmel, *D. subobscura*—Dsub, Kalna population—K, Slankamen population—Sl, standard (control) substrate—St, Pb-saturated substrate—C3, males—M, females—F and larvae—L.

### 2.3. Statistical Analysis of Life History Traits

Prior to the analyses of life history traits, all data were tested for normality by the Shapiro-Wilk test. Since the data for both egg-to-adult viability and egg-to-adult developmental time were normally distributed, we used the three- and four-factor ANOVA test to ascertain statistical significances. For egg-to-adult developmental time, the analyses were performed for males and females separately. The Bonferroni post-hoc test was used to identify the exact statistically significant differences. All the tests were performed in Statistica, ver. 10.0.228.2 [20].

### 2.4. Total DNA Extraction and 16S rRNA Sequencing

Total DNA was extracted from pools of 25 males, 25 females and 40 larvae for each experimental group, with 24 samples in total. DNA isolation was performed according to the modified protocol by Kapun et al. [21]. The samples were homogenized using handheld motor homogenizer in a 1.5 mL Eppendorf tube in 300 µL of Solution A (1 M Tris HCl, pH 9, 0.5 M EDTA, pH 8 and 1% SDS). Then 4.5 µL of Proteinase K (20 mg/mL) were added, and the samples were vortexed and incubated for 30 min at 56 °C, after which they were incubated for another 30 min at 70 °C and, finally, for a few minutes at 37 °C. Afterwards, 3 µL of RNAse A (10 mg/mL) were added and incubated for 30 min at 37 °C. Next, 42 µL 8 M potassium acetate was added and the mixture was kept in the freezer for 30 min. The supernatant was collected after centrifugation at 13,000 rpm for 15 min. One volume of phenol-chloroform-isoamyl alcohol (25:24:1) was added and the mixture was centrifuged for 5 min at 13,000 rpm. After the supernatant was collected, the previous step was repeated with 0.75 volume of pure chloroform. Afterwards, 2.5 volume of 95% ice-cold ethanol was added and centrifuged at 10,000 rpm for 5 min to pellet the DNA. The pellet was washed with 1 mL of 70% ethanol, centrifuged at 13,000 rpm for 5 min and then the supernatant was discarded. The pellet was dried for 30 min and left to resuspend in 50 µL of TE buffer. DNA quality was assessed using the Qubit fluorometer (Thermo Fisher Scientific, Waltham, MA, USA).

The sequencing was performed by Eurofins Genomics Europe Sequencing GmbH (Konstanz, Germany) using the standard procedure InView—Microbiome Profiling 3.0 with MiSeq. The V3-V4 hypervariable region of the 16S rRNA gene was amplified using the forward primer 5′-TACGGGAGGCAGCAG-3′ and reverse primer 5′-CCAGGGTATCTAATCC-3′ [22,23]. The samples passed a quality check and fastq data were delivered for further processing. All sequence data were submitted to the GenBank (SRA) database under accession numbers SRX12591286-SRX12591309 (PRJNA616141 BioProject).

### 2.5. Amplicon Sequence Variant Inference and Taxonomy Assignment

The delivered sequences already had the adapters and linkers removed. The primers were removed using cutadapt 3.4 (https://cutadapt.readthedocs.io/en/stable/guide.html, accessed on 2 December 2021) in paired-end mode and reads without detected primers were discarded. Afterwards, dada2 [24] was used to filter and trim the reads. Apart from the default filter and trim options, the forward reads were trimmed to 270 bases while the reverse reads were trimmed to 200 bases and all the reads shorter than 150 bases were removed, as well as all those which had more than two estimated errors for the forward or reverse reads. After error estimation and dereplication, denoising was performed in selfConsist mode (the algorithm alternated between sample inference and error rate estimation until convergence). Sequence pair merger was performed with a minimal overlap of 20 bases without mismatches. Chimeric sequences were removed using default options in removeBimeraDenovo (https://rdrr.io/bioc/dada2/man/removeBimeraDenovo.html, accessed on 2 December 2021). After the inspection of amplicon sequence variants (ASV) length distribution, all ASV with a length between 400 and 428 were kept. Taxonomy assignment up to the genus level was performed with IDTAXA algorithm [25] using default parameters and the SILVA v138 database (https://www.arb-silva.de/documentation/release-138/, accessed on 2 December 2021). Species level classification was performed with a SILVA species assignment train set (https://zenodo.org/record/3731176, accessed on 2 December 2021) using exact sequence matching without mismatches; multiple matches were allowed (multiple species output). One ASV which had low read counts was not classified at the Kingdom level and was removed from further processing.

### 2.6. Microbiome and Statistical Analysis

Sequence diversity within samples (alpha diversity) was estimated using the phyloseq 1.36.0 R package [26] at the ASV level after rarefaction to even depth (sample with the lowest number of reads) and shown through estimators Shannon, Gini-Simpson and invSimpson indices. Observed and estimated richness was determined according to the number of observations (Observed) and the Chao1 index. Comparison of alpha diversity among *D. melanogaster* and *D. subobscura* samples, as well as the impact of the growth substrate on Chao1 and Shannon indices within each species, was determined using the Wilcoxon Rank-Sum test. For further analysis, prevalence filtering was performed.

For beta diversity and differential abundance estimation between samples, genus level aggregation was used (reads from ASV classified as the same genus were aggregated). Since many low occurrence genera were present in only one or a few samples, prevalence filtering was performed where all genera present in less than four samples were removed. Beta diversity was estimated using Double Principal Coordinate Analysis (DPCoA) [27] of the prevalence filtered data after rarefaction to even depth. Since DPCoA relies also on phylogenetic distances of sequences apart from abundances, a maxim likelihood (GTR+G(4)+I) phylogenetic model was estimated using a multiple sequence alignment of the microbial 16S sequences constructed to take into account RNA secondary structures. To assess if the microbial composition differs among species and among fixed effects (sex and substrate) within species, permutational multivariate analysis of variance (PERMANOVA) using distance matrices via R package vegan 2.5-7 [28] was performed on the DPCoA distance matrices.

To perform differential abundance estimation, a Wilcoxon Rank-Sum test was used to test for differences between the median relative abundances of each genus in each *Drosophila* species. *p*-value adjustment for multiple comparisons was performed according to the Benjamini-Hochberg method [29]. In addition to this, similar tests were performed to check if sex, substrate or population affect abundance of taxa within each species; however, in all cases no taxa were found significant with the nonparametric test. The R package metacoder 0.3.5 [30] was used to create a heat tree which visualizes taxonomic categories significantly differing among groups. The analyses were performed using the R (version 4.1) [31].

## 3. Results

### 3.1. Life History Traits Analysis

We investigated the influence of the composition of microbiota, different feeding substrates and population origin on two *Drosophila* life history traits, egg-to-adult viability and developmental time. Mean values revealed that egg-to-adult viability was the highest in Dmel_K_St (0.88) and the lowest was in Dmel_Sl_C3 (0.51), while developmental time was the highest in Dsub_Sl_C3_M (23.06) and the lowest was in Dmel_K_St_F (19.24) (Table 1).

The analysis of different effects showed that population origin, substrate, species and sex were significant for differences in both life history traits, but when it comes to the interactions between these factors, not all interactions were significant (Table 2).

**Figure 1:**
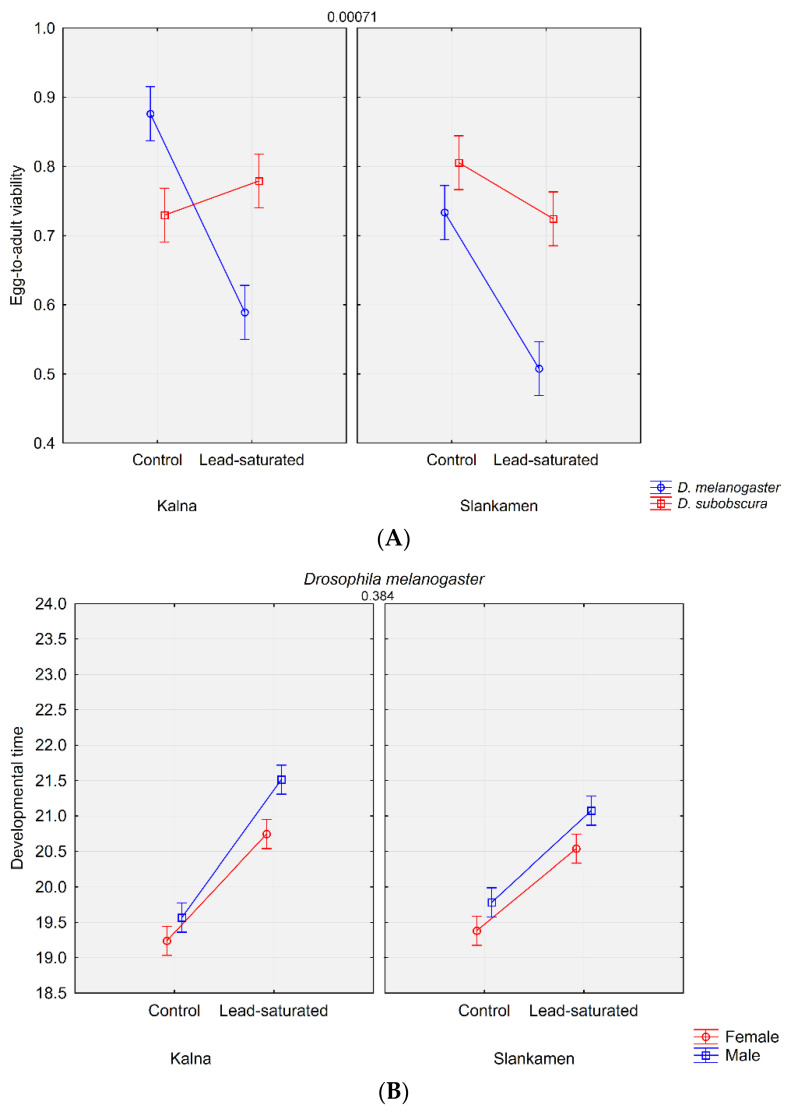

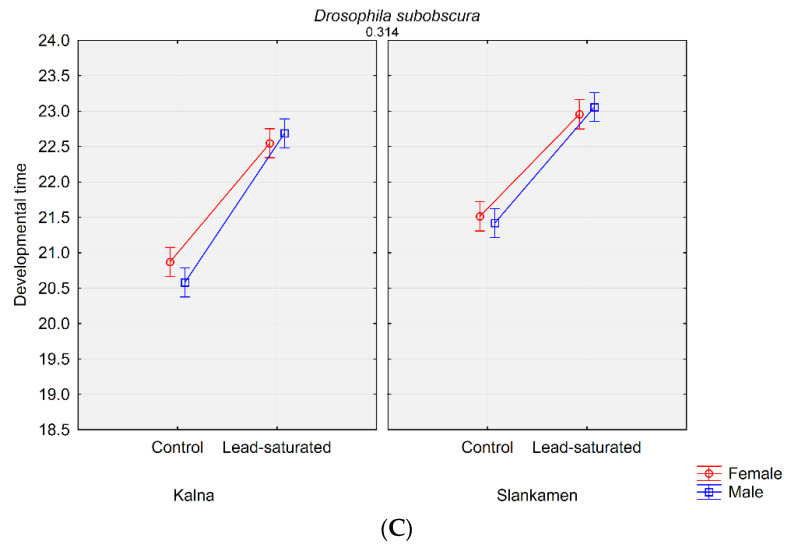
The LS mean plots of the analyzed traits depict changes that have occurred in different groups and subgroups; (**A**) egg-to-adult viability; (**B**) developmental time in *D. melanogaster;* (**C**) developmental time in *D. subobscura*. The numbers above the graphs indicate the *p*-values of the comparisons. The modified figure (**A**) was presented as a poster presentation at IECE2021 conference.

The post-hoc analysis revealed that egg-to-adult viability did not differ significantly between populations in *D. subobscura* on both the standard and the lead-saturated substrate. There were also no significant differences between the substrates within either population for *D. subobscura*. *D. melanogaster* showed no significant differences between the populations on the lead-saturated substrate (Figure 1A). All other interactions showed statistically significant differences (*p* < 0.05) in comparisons.

The results obtained for developmental time included sex as another potential factor that could be differentially affected regarding the population, species and substrate. The post-hoc analysis revealed that, on the standard substrate, there were no significant differences between males and females for both species and both populations. *D. subobscura* showed this pattern on the lead-saturated substrate for both populations as well, while in *D. melanogaster* sex differences were significant on the lead-saturated substrate for both populations. In *D. melanogaster*, both sexes from the same substrate but different populations did not differ significantly (Figure 1B). In *D. subobscura*, this pattern was observed only for lead-saturated substrate (Figure 1C). All other interactions showed statistically significant differences (*p* < 0.05) in comparisons.

### 3.2. Taxonomic Abundance of Microbial Communities

A total of 2,706,363 reads in 24 samples were obtained after quality filtering and these were used for ASV inference (Supplementary Table S1). All but one ASV were classified up to the order level (100 ASVs), 81 ASVs were classified up to the genus level and 49 to the species level (Supplementary Tables S2–S4). One ASV could not be assigned to Bacteria, and since it was present in traces only in one sample it was removed from further analysis.

**Figure 2:**
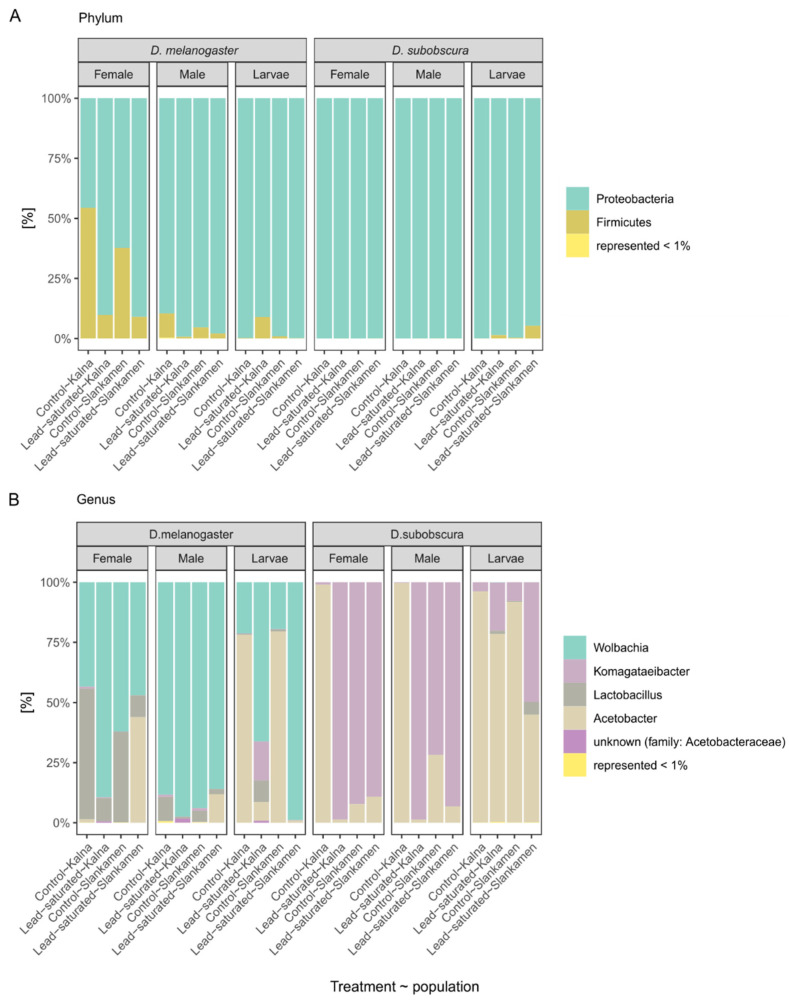
Relative abundance of the most prevalent (**A**) phyla and (**B**) genera in *Drosophila* species (*D. melanogaster* and *D. subobscura*) from two populations (Kalna and Slankamen) on the control substrate and the lead-saturated substrate in larvae and adult males and females. The modified figure (**B**) was presented as a poster presentation at IECE2021 conference.

The microbiota of two *Drosophila* species, *D. melanogaster* and *D. subobscura,* was classified into six phyla. At the phylum level, the most abundant phyla in total were Proteobacteria and Firmicutes with 86.6% and 13.3%, respectively; other four phyla accounted for less than 1% in total (Figure 2A).

Phylum Proteobacteria was dominant in almost all samples (23 of 24). On the other hand, Firmicutes was highly represented only in *D. melanogaster*, while its prevalence in *D. subobscura* was less than 2% out of total Firmicutes. Phylum Firmicutes was 99.9% represented by the genus *Lactobacillus*. Other most prevalent genera in all the samples were *Wolbachia, Komagataeibacter* and *Acetobacter* (Figure 2B). *Wolbachia* (36.9%) was present in all the samples of *D*. *melanogaster* flies, ranging from 2% to 21.6%, while it was present only in traces in two *D*. *subobscura* samples (<0.01%). The second most represented genus was *Komagataeibacter* (25%), with 95.9% found in *D. subobscura* samples. The increase in abundance of *Komagataeibacter* on the lead-saturated substrate was observed in five of six comparisons. Another highly represented genus from the Acetobacteraceae family was *Acetobacter*, with 65.5% in *D. subobscura* and 33.5% in *D. melanogaster* samples. Within each group, *Acetobacter* was the most prevalent in larvae samples. Another genus represented by >10% in total was *Lactobacillus* (13.3%), with 98.2% found in *D. melanogaster* samples, with higher prevalence in adults. All other genera (such as *Vibrionimonas, Staphylococcus, Sphingomonas* and *Acinetobacter*) were abundant with less than 1% in total. In *D. subobscura* samples, rare genera such as *Staphylococcus, Sphingomonas, Vibrionimonas, Acinetobacter* and a genus from family Xanthobacteraceae were present only in larvae samples. Interestingly, in *D. melanogaster* samples, *Staphylococcus, Sphingomonas* and *Vibrionimonas* were completely absent from larvae samples, but also from all lead-saturated substrate samples.

### 3.3. Alpha Diversity Analysis

Alpha diversity was measured using several metrics: Observed, Chao1, Shannon, Gini-Simpson and invSimpson indices (Table 3).

**Figure 3:**
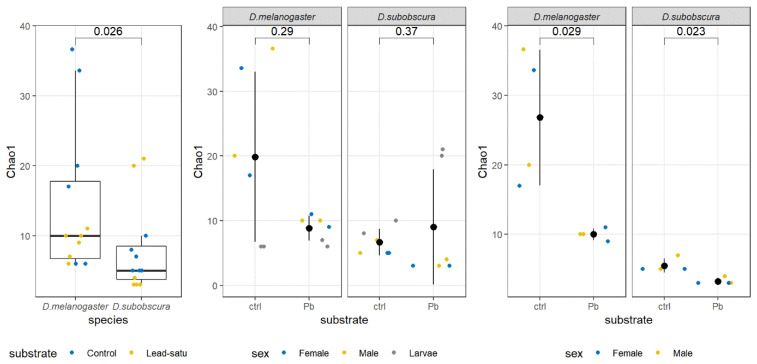
Differences in the Chao1 index between the species and the treatments estimated with the Wilcoxon Rank-Sum test. The number above the data indicates the *p*-value of the comparison.

In terms of alpha diversity, the Shannon index did not show statistically significant differences among groups of samples, but the Chao1 estimator was significantly lower in *D. subobscura* samples (Figure 3). When control versus lead substrate samples were compared within each of the species, no significant differences in Chao1 were observed in any of the samples included. However, if larvae samples were excluded, the Wilcoxon Rank-Sum test indicated a significantly lower Chao1 index in lead substrate samples for both species (Figure 3).

According to the Shannon and Simpson diversity indices, the highest diversity in adults was observed within the *D. melanogaster* female samples from the Kalna (standard) and from Slankamen (standard and lead-saturated substrate). Regarding the larvae samples, the highest diversity was observed in *D. melanogaster*, the Kalna population on lead and both *D. subobscura* samples on lead (Kalna and Slankamen). The lowest diversity in adults was found in both sexes and from both substrates in *D. subobscura* from Kalna population. Larvae samples with the lowest diversity were *D. melanogaster* from Slankamen on lead and *D. subobscura* from the Kalna population on the standard.

Similar to the Shannon and Simpson diversity indices, observed richness and Chao1 estimators were highest in adult *D. melanogaster* samples on the standard in the Kalna population, both sexes and the Slankamen population on the standard in males. The lowest richness in adults was observed in seven out of eight *D. subobscura* samples. Larvae richness was higher in *D. subobscura* on lead (both population) than in all *D. melanogaster* larvae samples.

### 3.4. Beta Diversity Analysis

**Figure 4:**
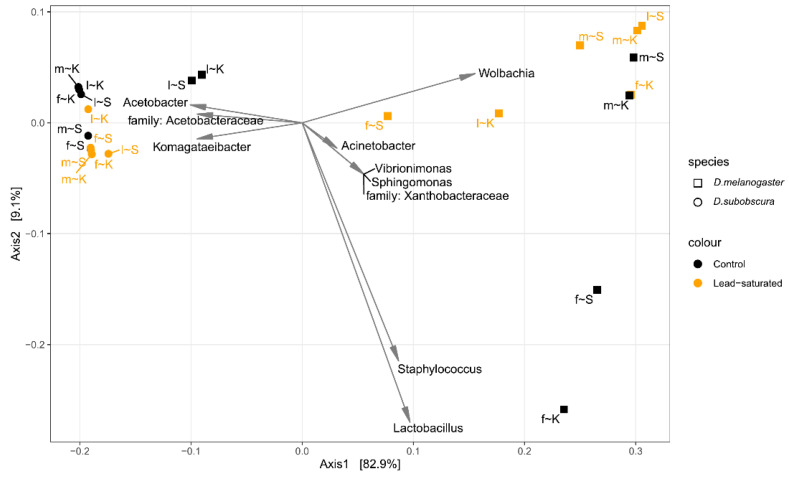
Biplot of Double Principal Coordinate Analysis (DPCoA) of bacterial composition in different *Drosophila* samples (*D. melanogaster* and *D. subobscura*; control and lead-saturated substrate indicated in the legend) using prevalence filtered taxa at the genus level aggregation. f—female; m—male; l—larvae; S—Slankamen population; K—Kalna population.

Beta diversity was estimated using Double Principal Coordinate Analysis (DPCoA) on prevalence filtered taxa at the genus level aggregation after rarefication to even depth (Figure 4).

Samples belonging to the two *Drosophila* species were separated by the first DPCoA axis. All *D. subobscura* samples clustered together, while *D. melanogaster* samples were dispersed on the biplot. Partial separation between the control substrate and the lead-saturated substrate of *D. subobscura* samples was observed by the second DPCoA axis. In addition, the larvae from both control *D. melanogaster* populations were positioned close to *D. subobscura* samples.

To investigate the source of variation in microbial composition, or more precisely to partition it among fixed effects such as species, sex, substrate and their interaction, PERMANOVA using DPCoA distances among the samples was performed on all samples and separately for *D. melanogaster* and *D. subobscura* sample subsets. When DPCoA distances among all samples are taken into account, the greatest source of variation was due to species and all interactions were significant (Table 4). When *D. melanogaster* samples were analyzed separately, substrate, sex and sex × substrate interaction were statistically significant (*p* < 0.05), indicating that the substrate had a different effect depending on the sex. For the *D. subobscura* samples DPCoA distance matrix, the substrate, sex and sex × substrate interaction did not have a significant effect.

**Figure 5:**
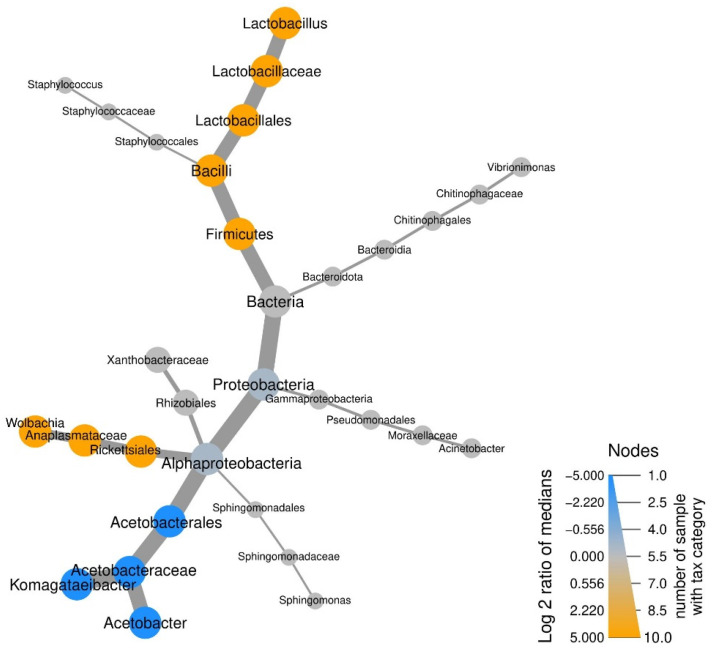
Differential heat tree of microbial communities’ abundances in *Drosophila* species with adjusted *p*-values < 0.05. Orange-colored taxa are more abundant in *D. melanogaster* while blue-colored taxa are more abundant in *D. subobscura*. The size of the circle indicates the number of samples with taxonomic category.

Differential abundances of the most prevalent genera indicate the dominance of the Acetobacteraceae family in *D. subobscura* samples, whereas the Lactobacillaceae family was predominant in *D. melanogaster* (Figure 5). Substrate and sex had no effect if all samples were taken into account, nor when *D. subobscura* and *D. melanogaster* were analyzed separately.

## 4. Discussion

In this study we observed that the overall bacterial diversity and richness were higher in adult *D. melanogaster* compared to *D. subobscura* samples. Life history analysis showed significant differences in population origin, substrate, species and sex effects on egg-to-adult viability and developmental time. The most prevalent genera in all the samples were *Wolbachia, Komagataeibacter*, *Acetobacter* and *Lactobacillus*. The genus *Lactobacillus* was dominantly abundant in *D. melanogaster* species on the standard substrate, while *Komagataeibacter* genus was dominant in *D. subobscura* on lead-saturated substrate. *Komagataeibacter* genus proved to be a species-specific member of *D. subobscura* microbiota that could be beneficial in overcoming environmental stress.

Comparing the results obtained from the composition of microbiota and life history traits of two *Drosophila* species reared on different substrates, several potential cause-and-effect relationships were discovered. The overall microbial diversity and evenness in *D. subobscura* reared in the laboratory was lower than in *D. melanogaster*, as in our previous study which was done only on the Kalna population [5]. With the addition of the Slankamen population in this research it can be seen that the Kalna population in *D. melanogaster* was mainly advantageous regarding the microbial diversity and richness compared to the Slankamen population. Contrary to that, *D. subobscura* originating from the Slankamen population showed higher diversity than the Kalna population. A similar pattern was observed for both adults and larvae, which indicates that the change of microbiota diversity due to the lead exposure could be population-specific. Microbial richness estimated by the Chao1 estimator was lower in *D. subobscura* in overall analysis, but also in both species on the substrate saturated with lead, when only males and females were included. Beta diversity showed that the differences in bacterial diversity were most expressed on the species level, but also revealed that microbial composition of *D. melanogaster* larvae was more similar to *D. subobscura* adult samples than to *D. melanogaster*. *D. subobscura* samples showed strong clustering and indicated a minor impact of lead exposure to variation in microbial composition. This could be due to the increase in the *Komagataeibacter* genus within the lead-saturated samples, which has been reported as a good probiotic candidate due to its high level of glucose conversion rate and survival rate in the presence of acidic pH and bile salt [32].

Life history traits also varied significantly between the populations and kept a similar pattern as microbiota in *D. melanogaster* on the standard substrate, whereby egg-to-adult viability was higher in the Kalna population compared to Slankamen. *D. subobscura* did not show significant differences in egg-to-adult viability regarding the origin, nor regarding the substrate composition, as was shown by Tanasković et al. [17]. On the other hand, developmental time results revealed that *D. subobscura* underwent more changes in this trait regarding the microbial composition, population origin and substrate than *D. melanogaster*. Namely, developmental time varied significantly between *D. subobscura* males and females on the standard substrate in both populations, but also within each sex on the standard substrate with different population origin. Males took longer to develop on the standard substrate compared to females, but both males and females originating from the Slankamen population took longer to develop than the Kalna population. This could be due to pre-adaptation to the polluted environment, as Kalna is probably more polluted area compared to Slankamen. *D. melanogaster* did not show significant differences in developmental time regarding the origin, nor regarding the substrate composition. This indicates that in some species, specific traits could be more susceptible to lead toxicity and changes in microbiota than others.

Microbial composition analysis indicated the dominance of the Acetobacteraceae family in *D. subobscura* samples and the Lactobacillaceae family in *D. melanogaster* species. Thus, *D. melanogaster*, the sample with the highest diversity and richness in microbiota species and the highest representation of genus *Lactobacillus*, showed the highest egg-to-adult viability and the shortest developmental time (Dmel_K_St, females), while the sample which showed microbial diversity albeit poor richness and domination by the genus *Acetobacter* exhibited lower egg-to-adult viability (Dmel_Sl_C3, females). Additionally, it was observed that in larvae of this species, the genus *Acetobacter* was dominant on the standard substrate, while its presence was significantly lower on the substrate with lead. *Lactobacillus* and *Acetobacter* are commonly found in lab-reared *D. melanogaster* [4,33,34]. Both genera can promote growth via different pathways, and in certain conditions, the presence of *Lactobacillus plantarum* helps larval growth and reduces their developmental time, so this could be a reason for short developmental time in Dmel_K_St [35,36,37]. The presence of 98.2% of total *Lactobacillus* ASVs in *D. melanogaster* suggested that *Lactobacillus* could be a species-specific member of *D. melanogaster* gut microbiota. The *Acetobacter* genus was present in larvae samples of both species, with the highest abundance on the standard substrate, but there was a decrease in egg-to-adult viability when *Acetobacter* was accumulated by adults (Dmel_Sl_C3 and Dsub_K_St). Previous studies have shown that lead toxicity can drive oxidative stress in many organisms [38,39,40,41,42]. Oxidative stress in *D. melanogaster* can cause various health defects, including reduced lifespan, retarded development, decreased pupation, emergence and survival rates, impaired mobility and reduced egg production [38,43,44,45]. In addition to the fact that lead has been proven to affect various life history traits, highly reduced viability in *D. melanogaster* on the lead-saturated substrate compared to *D. subobscura* could be a cost of faster development [46,47] and presence of endosymbiotic bacteria (*Wolbachia*), which also have been confirmed to affect the gut microbiota [48,49]. Short developmental time tends to have various fitness costs besides the reduced egg-to-adult viability, such as lower pathogen resistance [50], borderline larval storage of metabolites and reduced adult size [47]. Prolonged developmental time could be a potential mechanism of resistance to heavy metal exposure, providing a higher egg-to-adult viability.

Another possible factor that is greatly involved in shaping the microbiota is temperature [51]. *D. melanogaster* is successfully reared at 19–25 °C, unlike *D. subobscura,* which has a much tighter temperature range in the wild; 19 °C is the optimal rearing temperature in the lab. Heat-stressed *D. subobscura* flies showed changes in bacterial diversity and structure compared to non-stressed flies, and this response demonstrates that the gut microbiota contributes to heat tolerance, which could have important consequences on host fitness [52]. The sub-optimal rearing temperature for *D. melanogaster* could affect the metabolic strategy during the development, but also the growth of the species-specific microbiota. Additional experimental temperature manipulation would probably give a more complete answer in that sense.

Although the *D. subobscura* showed lower richness in microbiota species and lower diversity (Kalna population, both sexes and both substrates), the samples that were predominantly represented by the genus *Komagataeibacter* mainly maintained similar levels of egg-to-adult viability. *D. subobscura* samples from standard substrate, where *Komagataeibacter* genus was highly abundant (Slankamen population), also showed an increase in egg-to-adult viability compared to the population with low prevalence of *Komagataeibacter*. This indicates that the high prevalence of the genus *Komagataeibacter* was beneficial for flies’ viability in lab-rearing conditions, but also that it could be the key to the higher tolerance to lead exposure in *D. subobscura*. We previously reported the increase of *Komagataeibacter* in lab-reared flies after 13 generations, where its abundance drastically increased on the lead-saturated substrate, pointing to its higher tolerance to this heavy metal if compared to the other members of the microbial community [5]. After 35 generations, its prevalence has been maintained on the lead-saturated substrate, indicating a good heavy metal adaptation of *D. subobscura* species [15]. Measuring of the concentration of lead by inductively coupled plasma optical emission spectrometry (ICP-OES) in *D. melanogaster* and *D. subobscura* flies maintained for more than 30 generations in the control and lead-saturated substrate conditions showed that *D. subobscura* flies on lead-saturated substrate accumulated more lead than *D. melanogaster* (unpublished data). Moreover, the amount of lead accumulated was higher in males than in females in *D. subobscura*, whereas in *D. melanogaster* it was the opposite. The resistance of *D. subobscura* to increased accumulation of lead could be due to the prevalence of the *Komagataeibacter* genus, which could be an example of stable gut-colonizing bacteria in *D. subobscura*, since it has been proven to have a strong anti-oxidant ability in vitro [53] and it is considered to be a good probiotic candidate [32].

Taxonomic analysis revealed that the rare genera (<1%) in *D. subobscura* were present only in larvae, whereby *Staphylococcus* and *Acinetobacter* were present on the lead-saturated substrate, and *Sphingomonas, Vibrionimonas* and a genus from the family Xanthobacteraceae were present in larvae from both substrates. Interestingly, in *D. melanogaster* most of them were almost completely absent in larvae from both substrates, but also in the majority of adults from the lead-saturated samples. These findings suggest a different dynamic of developmental stages, as well as variability in substrate utilization and degradation by larvae in two species. These implicate the modulation in adaptive strategies under different environmental conditions in the two species.

## 5. Conclusions

In this study, we observed different patterns of life history traits in accordance with population origin and sex, but also the dominance of different gut microbiota members. The population origin showed a significant influence on life history traits, though each of the traits in the two species was affected differentially. Sex differences were also expressed, but only in *D. subobscura* on the standard substrate, indicating that influence of population origin and sex on life history traits could be species-specific. The presence of the heavy metal caused shifts in developmental time in *D. subobscura*, but maintained the egg-to-adult viability at a similar level. This could be explained by the domination of the *Komagataeibacter* in *D. subobscura* gut microbiota, usually a rare member of the microbiota community. The egg-to-adult viability increased in *D. subobscura* on standard substrate when *Komagataeibacter* was highly abundant, indicating that it could be a valuable member of *D. subobscura* microbiota in overcoming environmental stress. Research of the impact of microbiota on the adaptive response to heavy metals and the potential implications on host fitness is of great importance. Further research could reveal the extent to which species, sex, origin, lead exposure and specific members of microbiota, individually or through interactions, affect the life history traits. It could also help to identify the exact members of the gut microbiota that enable the best possible response to a particular environmental change.

## Figures and Tables

**Table 1:** Mean values of egg-to-adult viability and developmental time in all samples.

Sample	Egg-to-Adult Viability	Developmental Time
	Mean ± SE	Mean ± SE
Dmel_K_St_M	0.8763 ± 0.0068	19.567 ± 0.0482
Dmel_K_St_F		19.238 ± 0.0578
Dmel_Sl_St_M	0.7333 ± 0.0180	19.782 ± 0.0727
Dmel_Sl_St_F		19.381 ± 0.0588
Dmel_K_C3_M	0.5890 ± 0.0270	21.513 ± 0.1788
Dmel_K_C3_F		20.744 ± 0.1617
Dmel_Sl_C3_M	0.5077 ± 0.0298	21.075 ± 0.1359
Dmel_Sl_C3_F		20.537 ± 0.1727
Dsub_K_St_M	0.7297 ± 0.0166	20.580 ± 0.0593
Dsub_K_St_F		20.870 ± 0.0503
Dsub_Sl_St_M	0.8053 ± 0.0165	21.420 ± 0.0715
Dsub_Sl_St_F		21.514 ± 0.0795
Dsub_K_C3_M	0.7790 ± 0.0180	22.687 ± 0.0913
Dsub_K_C3_F		22.547 ± 0.0859
Dsub_Sl_C3_M	0.7243 ± 0.0164	23.058 ± 0.1088
Dsub_Sl_C3_F		22.955 ± 0.0957

Dmel—*Drosophila melanogaster*; Dsub—*Drosophila subobscura*; K—Kalna population; Sl—Slankamen population; St—standard (control) substrate; C3—lead-saturated substrate; M—male; F—female.

**Table 2:** Results of the factorial ANOVA test on (a) egg-to-adult viability and (b) developmental time for different effects.

Trait	Effect	df	SS	MS	F	*p*
(a) egg-to-adult viability	Population	1	0.1550	0.1550	13.21	0.000343
	Substrate	1	1.1125	1.1125	94.79	0.000000
	Species	1	0.4133	0.4133	35.22	0.000000
	Population × Substrate	1	0.0177	0.0177	1.51	0.220906
	Population × Species	1	0.2257	0.2257	19.23	0.000018
	Substrate × Species	1	0.8688	0.8688	74.03	0.000000
	Population × Substrate × Species	1	0.1382	0.1382	11.78	0.000709
(b) developmental time	Population	1	7.3	7.3	22.3	0.000003
	Substrate	1	305.5	305.5	931.2	0.000000
	Species	1	356.7	356.7	1087.1	0.000000
	Sex	1	6.7	6.7	20.6	0.000007
	Population × Substrate	1	5.5	5.5	16.7	0.000052
	Population × Species	1	12.2	12.2	37.2	0.000000
	Substrate × Species	1	1.7	1.7	5.3	0.022203
	Population × Sex	1	0.0	0.0	0.0	1.000000
	Substrate × Sex	1	2.7	2.7	8.3	0.004196
	Species × Sex	1	8.9	8.9	27.1	0.000000
	Population × Substrate × Species	1	0.2	0.2	0.5	0.476053
	Population × Substrate × Sex	1	0.5	0.5	1.6	0.199702
	Population × Species × Sex	1	0.2	0.2	0.6	0.445763
	Substrate × Species × Sex	1	0.0	0.0	0.0	0.906872
	Population × Substrate × Species × Sex	1	0.0	0.0	0.0	0.869297

The modified table was presented as a poster presentation at IECE2021 conference.

**Table 3:** Alpha diversity of ASVs represented in the microbial community of *Drosophila*.

Sample	Observed	Chao1	se.chao1	Shannon	Gini-Simpson	InvSimpson
Dmel_K_St_M	36	36.6	1.18	0.57	0.21	1.27
Dmel_K_St_F	28	33.6	5.34	1.29	0.64	2.76
Dmel_Sl_St_M	20	20	0	0.34	0.12	1.13
Dmel_Sl_St_F	16	17	2.29	1.17	0.56	2.29
Dmel_K_C3_M	10	10	0	0.14	0.05	1.05
Dmel_K_C3_F	11	11	0	0.46	0.20	1.24
Dmel_Sl_C3_M	10	10	0	0.49	0.24	1.31
Dmel_Sl_C3_F	9	9	0	1.07	0.58	2.41
Dsub_K_St_M	5	5	0	0.05	0.02	1.02
Dsub_K_St_F	5	5	0	0.11	0.03	1.04
Dsub_Sl_St_M	7	7	0.46	0.60	0.41	1.69
Dsub_Sl_St_F	5	5	0.22	0.29	0.15	1.17
Dsub_K_C3_M	3	3	0	0.07	0.03	1.03
Dsub_K_C3_F	3	3	0	0.08	0.03	1.03
Dsub_Sl_C3_M	4	4	0	0.25	0.13	1.15
Dsub_Sl_C3_F	3	3	0	0.35	0.20	1.25
Dmel_St_K_L	6	6	0	0.56	0.35	1.53
Dmel_St_Sl_L	6	6	0.46	0.55	0.33	1.48
Dsub_St_K_L	8	8	0	0.18	0.08	1.09
Dsub_St_Sl_L	10	10	0.47	0.42	0.19	1.24
Dmel_C3_K_L	6	6	0.46	1.02	0.52	2.08
Dmel_C3_Sl_L	7	7	0	0.07	0.02	1.02
Dsub_C3_K_L	20	20	0	0.61	0.35	1.54
Dsub_C3_Sl_L	18	21	4.15	0.89	0.55	2.22

Dmel—*Drosophila melanogaster*; Dsub—*Drosophila subobscura*; K—Kalna population; Sl—Slankamen population; St—standard (control) substrate; C3—lead-saturated substrate; M—male; F—female; L—larvae.

**Table 4:** Results of PERMANOVA using DPCoA distances among all *Drosophila* samples.

Effect	df	SS	MS	F	R^2^	*p*
Species	1	0.893082	0.893082	132.2773	0.651498	0.001
Substrate	1	0.046581	0.046581	6.899272	0.033981	0.02
Sex	2	0.100009	0.050004	7.406304	0.072956	0.008
Species × Substrate	1	0.034041	0.034041	5.041911	0.024833	0.029
Species × Sex	2	0.08629	0.043145	6.390341	0.062948	0.013
Substrate × Sex	2	0.065261	0.032631	4.833024	0.047608	0.014
Species × Substrate × Sex	2	0.06453	0.032265	4.778869	0.047074	0.02
Residuals	12	0.081019	0.006752		0.059103	
Total	23	1.370812			1	

## Data Availability

The data presented in this study are available in Supplementary Materials.

## Supplementary Materials

The following are available online at http://www.mdpi.com/article/10.3390/insects12121122/s1, Table S1: Number of sequences before and after filtering, Table S2: Bacterial phylum taxonomy contingency table of microbiota in all samples, Table S3: Bacterial genus taxonomy contingency table of microbiota in all samples, Table S4: Bacterial species taxonomy contingency table of microbiota in all samples.

## References

[1] Zheng D., Liwinski T., Elinav E. (2020). Interaction between microbiota and immunity in health and disease. Cell Res..

[2] Dominguez-Bello M.G., Godoy-Vitorino F., Knight R., Blaser M.J. (2019). Role of the microbiome in human development. Gut.

[3] Morais L.H., Schreiber H.L., Mazmanian S.K. (2021). The gut microbiota–brain axis in behaviour and brain disorders. Nat. Rev. Microbiol..

[4] Wong C.N.A., Ng P., Douglas A.E. (2011). Low-diversity bacterial community in the gut of the fruitfly Drosophila melanogaster. Environ. Microbiol..

[5] Beribaka M.B., Dimkić I.Z., Jelić M.Đ., Stanković S.M., Pržulj N.M., Anđelković M.L., Stamenković-Radak M.M. (2021). Altered diversity of bacterial communities in two Drosophila species under laboratory conditions and lead exposure. Arch. Biol. Sci..

[6] Ryu J.-H., Kim S.-H., Lee H.-Y., Bai J.Y., Nam Y.-D., Bae J.-W., Lee D.G., Shin S.C., Ha E.-M., Lee W.-J. (2008). Innate Immune Homeostasis by the Homeobox Gene Caudal and Commensal-Gut Mutualism in Drosophila. Science.

[7] Iatsenko I., Boquete J.-P., Lemaitre B. (2018). Microbiota-Derived Lactate Activates Production of Reactive Oxygen Species by the Intestinal NADPH Oxidase Nox and Shortens Drosophila Lifespan. Immunity.

[8] Fast D., Duggal A., Foley E. (2018). Monoassociation with Lactobacillus plantarum disrupts intestinal homeostasis in adult Drosophila melanogaster. MBio.

[9] Lee H.-Y., Lee S.-H., Lee J.-H., Lee W.-J., Min K.-J. (2019). The role of commensal microbes in the lifespan of Drosophila melanogaster. Aging.

[10] Leitão-Gonçalves R., Carvalho-Santos Z., Francisco A.P., Fioreze G.T., Anjos M., Baltazar C., Elias A.P., Itskov P.M., Piper M.D.W., Ribeiro C. (2017). Commensal bacteria and essential amino acids control food choice behavior and reproduction. PLoS Biol..

[11] Jia Y., Jin S., Hu K., Geng L., Han C., Kang R., Pang Y., Ling E., Tan E.K., Pan Y. (2021). Gut microbiome modulates Drosophila aggression through octopamine signaling. Nat. Commun..

[12] Silva V., Palacios-Muñoz A., Okray Z., Adair K.L., Waddell S., Douglas A.E., Ewer J. (2021). The impact of the gut microbiome on memory and sleep in Drosophila. J. Exp. Biol..

[13] Clark R.I., Salazar A., Yamada R., Fitz-Gibbon S., Morselli M., Alcaraz J., Rana A., Rera M., Pellegrini M., Ja W. (2015). Distinct Shifts in Microbiota Composition during Drosophila Aging Impair Intestinal Function and Drive Mortality. Cell Rep..

[14] Monchanin C., Devaud J.-M., Barron A.B., Lihoreau M. (2021). Current permissible levels of metal pollutants harm terrestrial invertebrates. Sci. Total. Environ..

[15] Kalajdzic P., Kenig B., Andjelkovic M. (2015). Drosophila subobscura flies adapted to low lead concentration carry no fitness cost. Environ. Pollut..

[16] Kenig B., Stamenković-Radak M., Andelković M. (2013). Population specific fitness response of Drosophila subobscura to lead pollution. Insect Sci..

[17] Tanaskovic M., Novicic Z.K., Kenig B., Stamenkovic-Radak M., Andjelkovic M. (2015). Effect of lead pollution on fitness and its dependence on heterozygosity in Drosophila subobscura. J. Genet..

[18] Zhou S., Luoma S.E., St. Armour G.E., Thakkar E., Mackay T.F.C., Anholt R.R.H. (2017). A Drosophila model for toxicogenomics: Genetic variation in susceptibility to heavy metal exposure. PLoS Genet..

[19] Obadia B., Keebaugh E.S., Yamada R., Ludington W.B., Ja W.W. (2018). Diet influences host–microbiota associations in Drosophila. Proc. Natl. Acad. Sci. USA.

[20] StatSoft Inc. (2011). STATISTICA (Data Analysis Software System), version 10, 10.

[21] Kapun M., Barrón M.G., Staubach F., Obbard D.J., Wiberg R.A.W., Vieira J., Goubert C., Rota-Stabelli O., Kankare M., Bogaerts-Márquez M. (2020). Genomic Analysis of European Drosophila melanogaster Populations Reveals Longitudinal Structure, Continent-Wide Selection, and Previously Unknown DNA Viruses. Mol. Biol. Evol..

[22] Turner S., Pryer K.M., Miao V.P., Palmer J.D. (1999). Investigating deep phylogenetic relationships among cyanobacteria and plastids by small subunit rRNA sequence analysis. J. Eukaryot. Microbiol..

[23] Kisand V., Cuadros R., Wikner J. (2002). Phylogeny of culturable estuarine bacteria catabolizing riverine organic matter in the northern Baltic Sea. Appl. Environ. Microbiol..

[24] Callahan B.J., McMurdie P.J., Rosen M.J., Han A.W., Johnson A.J.A., Holmes S.P. (2016). DADA2: High-resolution sample inference from Illumina amplicon data. Nat. Methods.

[25] Murali A., Bhargava A., Wright E.S. (2018). IDTAXA: A novel approach for accurate taxonomic classification of microbiome sequences. Microbiome.

[26] McMurdie P.J., Holmes S. (2013). phyloseq: An R Package for Reproducible Interactive Analysis and Graphics of Microbiome Census Data. PLoS ONE.

[27] Pavoine S., Dufour A.-B., Chessel D. (2004). From dissimilarities among species to dissimilarities among communities: A double principal coordinate analysis. J. Theor. Biol..

[28] Oksanen J., Blanchet F.G., Friendly M., Kindt R., Legendre P., McGlinn D., Minchin P.R., O’Hara R.B., Simpson G.L., Solymos P. (2020). *Vegan: Community Ecology Package*, R package version 2.5-7. https://CRAN.R-project.org/package=vegan.

[29] Benjamini Y., Hochberg Y. (1995). Controlling the False Discovery Rate: A Practical and Powerful Approach to Multiple Testing. J. R. Stat. Soc. Ser. B.

[30] Foster Z.S.L., Sharpton T.J., Grünwald N.J. (2017). Metacoder: An R package for visualization and manipulation of community taxonomic diversity data. PLoS Comput. Biol..

[31] R Core Team (2021). R: A Language and Environment for Statistical Computing.

[32] Lavasani P.S., Motevaseli E., Sanikhani N.S., Modarressi M.H. (2019). Komagataeibacter xylinus as a novel probiotic candidate with high glucose conversion rate properties. Heliyon.

[33] Broderick N.A., Lemaitre B. (2012). Gut-associated microbes of Drosophila melanogaster. Gut Microbes.

[34] Chandler J.A., Lang J.M., Bhatnagar S., Eisen J.A., Kopp A. (2011). Bacterial Communities of Diverse Drosophila Species: Ecological Context of a Host–Microbe Model System. PLoS Genet..

[35] Storelli G., Defaye A., Erkosar B., Hols P., Royet J., Leulier F. (2011). Lactobacillus plantarum promotes Drosophila systemic growth by modulating hormonal signals through TOR-dependent nutrient sensing. Cell Metab..

[36] Shin S.C., Kim S.-H., You H., Kim B., Kim A.C., Lee K.-A., Yoon J.-H., Ryu J.-H., Lee W.-J. (2011). Drosophila microbiome modulates host developmental and metabolic homeostasis via insulin signaling. Science.

[37] Téfit M.A., Leulier F. (2017). Lactobacillus plantarum favors the early emergence of fit and fertile adult Drosophila upon chronic undernutrition. J. Exp. Biol..

[38] Liu Z.-H., Shang J., Yan L., Wei T., Xiang L., Wang H.-L., Cheng J., Xiao G. (2020). Oxidative stress caused by lead (Pb) induces iron deficiency in Drosophila melanogaster. Chemosphere.

[39] Martinez-Haro M., Green A.J., Mateo R. (2011). Effects of lead exposure on oxidative stress biomarkers and plasma biochemistry in waterbirds in the field. Environ. Res..

[40] Fan Y., Zhao X., Yu J., Xie J., Li C., Liu D., Tang C., Wang C. (2020). Lead-induced oxidative damage in rats/mice: A meta-analysis. J. Trace Elem. Med. Biol..

[41] Gurer-Orhan H., Sabır H.U., Özgüneş H. (2004). Correlation between clinical indicators of lead poisoning and oxidative stress parameters in controls and lead-exposed workers. Toxicology.

[42] Wang L., Wang H., Hu M., Cao J., Chen D., Liu Z. (2009). Oxidative stress and apoptotic changes in primary cultures of rat proximal tubular cells exposed to lead. Arch. Toxicol..

[43] Marcus S.R., Fiumera A.C. (2016). Atrazine exposure affects longevity, development time and body size in Drosophila melanogaster. J. Insect Physiol..

[44] Figueira F.H., Aguiar L.M.d., Rosa C.E.D. (2017). Embryo-larval exposure to atrazine reduces viability and alters oxidative stress parameters in Drosophila melanogaster. Comp. Biochem. Physiol. Part. C Toxicol. Pharmacol..

[45] Li F., Liu Z.-H., Tian X., Liu T., Wang H.-L., Xiao G. (2020). Black soybean seed coat extract protects Drosophila melanogaster against Pb toxicity by promoting iron absorption. J. Funct. Foods.

[46] Prasad N.G., Shakarad M., Gohil V.M., Sheeba V., Rajamani M., Joshi A. (2000). Evolution of reduced pre-adult viability and larval growth rate in laboratory populations of Drosophila melanogaster selected for shorter development time. Genet. Res..

[47] Chippindale A.K., Alipaz J.A., Chen H.-W., Rose M.R. (1997). Experimental evolution of accelerated development in drosophila. 1. Developmental speed and larval survival. Evolution.

[48] Simhadri R.K., Fast E.M., Guo R., Schultz M.J., Vaisman N., Ortiz L., Bybee J., Slatko B.E., Frydman H.M. (2017). The Gut Commensal Microbiome of Drosophila melanogaster Is Modified by the Endosymbiont Wolbachia. mSphere.

[49] Ye Y.H., Seleznev A., Flores H.A., Woolfit M., McGraw E.A. (2017). Gut microbiota in Drosophila melanogaster interacts with Wolbachia but does not contribute to Wolbachia-mediated antiviral protection. J. Invertebr. Pathol..

[50] Modak S.G., Satish K., Mohan J., Dey S., Raghavendra N., Shakarad M., Joshi A. (2009). A possible tradeoff between developmental rate and pathogen resistance in Drosophila melanogaster. J. Genet..

[51] Sepulveda J., Moeller A.H. (2020). The Effects of Temperature on Animal Gut Microbiomes. Front. Microbiol..

[52] Jaramillo A., Castañeda L.E. (2021). Gut Microbiota of Drosophila subobscura Contributes to Its Heat Tolerance and Is Sensitive to Transient Thermal Stress. Front. Microbiol..

[53] Jiang X., Lin D., Shao H., Yang X. (2019). Antioxidant properties of Komagataeibacter hansenii CGMCC 3917 and its ameliorative effects on alcohol-induced liver injury in mice. CyTA J. Food.

